# Anti-Tumor Immunity to Patient-Derived Breast Cancer Cells by Vaccination with Interferon-Alpha-Conditioned Dendritic Cells (IFN-DC)

**DOI:** 10.3390/vaccines12091058

**Published:** 2024-09-17

**Authors:** Caterina Lapenta, Stefano Maria Santini, Celeste Antonacci, Simona Donati, Serena Cecchetti, Patrizia Frittelli, Piera Catalano, Francesca Urbani, Iole Macchia, Massimo Spada, Sara Vitale, Zuleika Michelini, Domenico Cristiano Corsi, Ann Zeuner, Rosanna Dattilo, Manuela Tamburo De Bella

**Affiliations:** 1 Department of Oncology and Molecular Medicine, Istituto Superiore di Sanità, 00161 Rome, Italy; simona.donati@iss.it (S.D.); francesca.urbani@iss.it (F.U.); iole.macchia@iss.it (I.M.); sara.vitale@iss.it (S.V.); ann.zeuner@iss.it (A.Z.); rosanna.dattilo@iss.it (R.D.); 2 Department of Pediatric Hematology/Oncology and Cellular and Gene Therapy, Bambino Gesù Children’s Hospital, IRCCS, 00165 Rome, Italy; celeste.antonacci@opbg.net; 3 Core Facilities, Istituto Superiore di Sanità, 00161 Rome, Italy; serena.cecchetti@iss.it; 4 Medical Oncology Unit, Fatebenefratelli-Isola Tiberina Hospital, 00186 Rome, Italy; patrizia.frittelli@fbf-isola.it (P.F.); piera.catalano@fbf-isola.it (P.C.); domenico.corsi@libero.it (D.C.C.); 5 Center of Animal Research and Welfare, Istituto Superiore di Sanità, 00161 Rome, Italy; massimo.spada@iss.it; 6 National Center for Global Health, Istituto Superiore di Sanità, 00161 Rome, Italy; zuleika.michelini@iss.it; 7 Hospital Clinical Networks Governance e DM70/15 Monitoring—AGENAS—National Agency for Regional Health Services, 00187 Rome, Italy; tamburodebella@agenas.it

**Keywords:** cancer vaccines, immunotherapy, breast cancer, dendritic cells

## Abstract

Background: Breast cancer represents one of the leading causes of death among women. Surgery can be effective, but once breast cancer has metastasized, it becomes extremely difficult to treat. Conventional therapies are associated with substantial toxicity and poor efficacy due to tumor heterogeneity, treatment resistance and disease relapse. Moreover, immune checkpoint blockade appears to offer limited benefit in breast cancer. The poor tumor immunogenicity and the immunosuppressive tumor microenvironment result in scarce T-cell infiltration, leading to a low response rate. Thus, there is considerable interest in the development of improved active immunotherapies capable of sensitizing a patient’s immune system against tumor cells. Methods: We evaluated the in vitro anti-tumor activity of a personalized vaccine based on dendritic cells generated in the presence of interferon (IFN)-α and granulocyte-macrophage colony-stimulating factor (IFN-DC) and loaded with an oxidized lysate from autologous tumor cells expanded as 3D organoid culture maintaining faithful tumor antigenic profiles. Results: Our findings demonstrate that stimulation of breast cancer patients’ lymphocytes with autologous IFN-DC led to efficient Th1-biased response and the generation in vitro of potent cytotoxic activity toward the patients’ own tumor cells. Conclusions: This approach can be potentially applied in association with checkpoint blockade and chemotherapy in the design of new combinatorial therapies for breast cancer.

## 1. Introduction

Breast cancer (BC) represents the most common form of cancer among women, accounting for approximately 25% of all diagnosed cancers worldwide [1]. It represents a leading cause of death among women in the United States and Europe. The primary therapeutic approaches for BC encompass surgery, radiotherapy, chemotherapy and hormone therapy [2]. Notwithstanding the efficacy of trastuzumab and pertuzumab in treating HER2-positive BC, disease recurrence remains a significant issue for approximately 20% of patients [3]. There remains a paucity of targeted therapeutic options for patients who have become refractory to endocrine therapy, as well as those with triple-negative breast cancer (TNBC). Furthermore, conventional therapies are associated with considerable toxicity, prompting the development of new treatments to enhance therapeutic efficacy and mitigate undesirable side effects.

Although BC has historically been regarded as a poorly immunogenic malignancy, recent investigations have uncovered the important presence of tumor-infiltrating lymphocytes (TILs) in a number of BC tissues, associated with prognostic benefits [4,5,6]. An increasing body of evidence suggests that the immune system plays a pivotal role in determining responses to standard therapy and long-term survival in BC patients [7,8].

A number of tumor-associated antigens have been identified as potential targets for BC vaccination [9]. However, the heterogeneity of tumors and the potential loss of antigen expression over time may limit the efficacy of antigen-specific vaccines. An alternative approach involves the utilization of whole-tumor cell lysates from a patient’s own tumor as an antigen source, thereby triggering a broader immune response against both common and individual neoantigens [10]. In this context, the in vitro expansion of primary BC cells may provide a solution to the challenges associated with the collection of sufficient tumor cells for the completion of multiple vaccination cycles. Patient-derived tumor organoids provide a valuable alternative to traditional two-dimensional (2D) cell culture, maintaining the in vivo structure and faithful antigenic profiles. Furthermore, hypochlorous acid (HOCl)-mediated tumor cell oxidation has been demonstrated to enhance primary necrosis and lysate immunogenicity [10].

This study investigates the potential of monocyte-derived dendritic cells (IFN-DCs), generated in the presence of interferon-α (IFN-α) and granulocyte-macrophage colony-stimulating factor (GM-CSF) [11,12], and loaded with oxidized tumor cell lysate from patient-specific organoids to activate immune responses against BC cells. Our findings demonstrate the efficient induction of a Th1-type response, characterized by high levels of IFN-γ production and cytotoxic effector activity against autologous BC cells.

## 2. Materials and Methods

### 2.1. Patients and Samples

BC specimens and blood samples from were anonymously provided by the UOC Oncologia Ospedale S. Giovanni Calibita FBF Roma from patients undergoing surgical resection of primary tumors, paracentesis of ascitic fluid or thoracentesis of pleural effusion. Peripheral blood mononuclear cells (PBMC) from healthy donors were obtained from buffy coats anonymously provided by the Transfusional Center of Umberto I of Rome.

### 2.2. Statistical Analysis

No data normalization was performed before analysis. Statistical analyses were performed by Mann–Whitney or Wilcoxon test and Spearman’s rank correlation test, except where indicated. The mean ± standard error of the mean (SEM) was used to present the results.

### 2.3. Cell Lines

Breast cell line MCF-7 (Interlab Cell Line Collection) and human erythroblastoid cell line K562 (ATCC) were cultured in Dulbecco’s Modified Eagle Medium (DMEM) and Roswell Park Memorial Institute (RPMI) 1640 medium (Life Technologies, Carlsbad, CA, USA) supplemented with 10% fetal bovine serum FBS (for K562 cells) or 20% FBS (for MCF-7 cells), 2 mM l-glutamine, 100 U/mL penicillin and 100 μg/mL streptomycin. The HLA-A2 haplotype of MCF-7 cells was determined by fluorescence-activated cell sorting (FACS) analysis, using anti-A2–specific mAb (BD Biosciences, Franklin Lakes, NJ, USA).

### 2.4. Cell Preparation

IFN-DC were differentiated from CD14+ monocytes, positively selected by immunomagnetic sorting (MACS Cell Isolation Kit; Miltenyi Biotec, Bergisch Gladbach, Germany) and plated at 2 × 10^6^ cells/mL for 3 days in CellGro DC medium (CellGenix), supplemented with 500 U/mL GM-CSF (Genzyme) and 10,000 U/mL IFN-α2b (Intron A; Schering Plough), Comazzo, Italy). [11,13]. peripheral blood lymphocytes (PBL) were obtained from PBMC following CD14+ monocyte depletion.

### 2.5. Isolation of Patient-Derived BC Organoids (PDBCOs) from Primary Tumors

Specimens of primary tumors were processed as previously reported [14]. Recovered cells were plated as organoid cultures by following the method described by Clevers and collaborators [15]. BC organoid medium was changed every 4–7 days, and organoids were dissociated every 1–4 weeks by pipetting up and down or, for dense organoids, through an incubation with a mixture of TrypLE Select reagent (Thermo Fischer Scientific, Waltham, MA, USA) and Accumax solution (Sigma Aldrich, Burlington, MA, USA) for 1–2 min at 37 °C.

### 2.6. Isolation of Patient-Derived Metastatic (PDM) Cells from Ascitic Fluid or Pleural Effusion

Specimens of ascitic fluid or pleural effusion were centrifuged and the pellet was seeded as liquid culture in non-treated flasks (Corning Incorporated, Corning, NY, USA) in serum-free medium supplemented with  20 ng·ml^−1^ epidermal growth factor (EGF) (Peprotech, Cranbury, NJ, USA), 10  ng·ml^−1^ basic fibroblast growth factor (b-FGF) (PeproTech), 50 µg/mlinsulin (Sigma–Aldrich), 1× B-27 (Gibco–Invitrogen, Waltham, MA, USA) and 10% patient’s ascitic or pleural fluids previously filtered. The medium was replaced once a week.

### 2.7. Confocal Laser Scanning Microscopy (CLSM)

To perform CLSM analysis, organoids were left to grow directly on µ-Slide 8 Well plates (Ibidi, Gräfelfing, Germany), while liquid cultures samples were cytospinned on poly-lysine coated slides.

Organoids and cells were fixed respectively with 2 or 4% paraformaldehyde for 20 min, permeabilized with 0.1% Triton X-100 and stained with the following antibodies: β-Catenin (D10A8) XP^®^ Rabbit mAb, Vimentin (D21H3) XP^®^ Rabbit mAb, E-cadherin (24E10) Rabbit mAb (all from Cell Signaling, Danvers, MA, USA), Anti-alpha smooth muscle Actin antibody (1A4), Anti-Cytokeratin 8 + 18 antibody (5D3) (all from Abcam, Cambridge, UK), anti-TAZ Purified Mouse Clone M2-616 (RUO) (Bd Bioscience), followed by the appropriate secondary antibodies (Alexa Fluor, Invitrogen). Nuclei were detected by using DAPI (Invitrogen). CLSM observations were performed on a Zeiss LSM980 apparatus (Zeiss, Oberkochen, Germany). Cells stained only with the fluorochrome-conjugated secondary antibody were used to set up acquisition parameters. Several fields of view (>100 cells and >50 organoids) were analyzed for each labeling condition.

### 2.8. Flow Cytometry

Degranulation analysis was performed on day 14 of culture in order to detect CD107a ectopic expression activity together with intracellular IFN-γ production in response to cancer cells. PBL were incubated with patient-derived BC cells, MCF-7 or K562 target cells (effector-to-target (E:T) ratio of 2:1) in the presence of fluorescein-5-isothiocyanate (FITC) anti-CD107a (BD Biosciences and Golgi Plug (1 μg/mL; BD Biosciences) for 4 h. Tests were performed in V-bottom 96-well microtiter plates (Nunc) for 4 h at 37 °C. Cells were stained with anti CD3, CD8 and CD56 antibodies, fixed and permeabilized with BD Cytofix/Cytoperm Plus (BD Biosciences), then stained with PE-labeled anti-IFN-γ (BD Biosciences).

Cell acquisition and analysis were performed using either an LSRII (Becton Dickinson, Franklin Lakes, NJ, USA) or a Gallios (Beckman-Coulter Life Sciences, Indianapolis, IN, USA) flow cytometers.

### 2.9. Immunohistochemistry

Matrigel drops containing organoids were resuspended in cold PBSB (phosphate-buffered saline (PBS)-1× supplemented with 0.1% bovine serum albumin), transferred to a 15 mL centrifuge tube, and allowed to settle under gravity on ice for 30 min. Metastatic cells were collected by centrifugation. A 0.5 mL aliquot of plasma was added to each sample, vortexing briefly. Next, 0.5 mL of reconstituted thrombin was added and a clot was formed in 1–2 min. The clot was placed into a labeled cassette containing formalin. The specimen was then routinely processed.

### 2.10. Lysate Tumor Cell and PBL/DC Cocultures

To prepare oxidized lysates, MCF-7 and BC patient-derived cells were incubated with 300 μM HOCl in Dulbecco’s phosphate buffered saline (DPBS) for 15 min at 37 °C. Then, cells were collected, washed to remove any unreacted HOCl, and resuspended in DPBS at a density of 1 × 10^7^ cells per mL. Cells were subjected to five freeze–thaw cycles using dry ice and a 37 °C water bath and centrifuged. The soluble fraction was collected as the oxidized lysate. IFN-DC were pulsed with lysate tumor cells at a 1:2 ratio for 16 h at 37 °C and then cultured with autologous PBL at a 1:4 ratio. Medium was supplemented with human IL-2 (50 U/mL; Roche, Basel, Switzerland), starting from day 3 of coculture, once every 3 d. On day 7 of coculture, PBL were restimulated with frozen stocks of lysate cell-loaded DC, and the effector cells were analyzed 7 days later.

### 2.11. Therapeutic Vaccination of Tumor-Bearing Hu-PBL-NSG Mice

Animal procedures were performed according to the Italian national animal experimentation guidelines (D.L.26/2014) upon approval of the experimental protocol by the Italian Ministry of Health’s Animal Experimentation Committee (DM n.1158/2020-PR D9997.119). Six-week-old female NOD.Cg-Prkdcscid Il2rgtm1Wjl/SzJ mice (NSG, Tokyo, Japan) were purchased from Charles Rivers and injected subcutaneously (s.c.) in the hind flank with 4 × 10^6^ MCF-7 cells stably transfected as already described [16] with the pCH-CMV-eGFPWT2FflUC lentiviral vector expressing the eGFP and the luciferase gene. Mice were reconstituted with 5 × 10^6^ human PBL from HLA-A2+ healthy donors 11 days later and randomized into treatment and control groups. The resulting hu-PBL-NSG mice were vaccinated (i.p) on day 12 with 2 × 10^6^ IFN-DC loaded with 100 µg HOCl-oxidized MCF-7 cell lysate, while control hu-PBL-NSG mice received unloaded IFN-DC. Mice received boost immunizations on day 19 and day 26. Tumor growth was monitored for 47 days using a bioluminescence imaging system (IVIS 100 Imaging System, Xenogen, Alameda, CA, USA) following luciferin administration (i.p.) (100 mg/kg, Perkin Elmer, Waltham, MA, USA).

### 2.12. Cytokine Assay

Cytokine quantification was performed using commercial ELISA kits for IFN-γ (15.6 pg/mL) (Elabscience, Houston, TX, USA), TNF-α (15.6 pg/mL), IL-10 (3.9 pg/mL) (Diaclone, Besancon, France) and IL-12 (3.16 pg/mL) (Mabtech, Stockholm, Sweden) according to the manufacturers’ instructions.

### 2.13. Cytotoxicity Assay

Cytotoxicity was tested against MCF-7, K562 or patient-derived breast cells. We resuspended target cells in 15 μM Calcein-AM (Sigma–Aldrich) for 30 min at 37 °C, with occasional mixing. Cytotoxic activity was assessed using E:T ratios ranging from 10:1 to 0.6:1 in triplicate as previously described [17].

## 3. Results

### 3.1. In Vitro Induction of Immune Responses to MCF-7 Breast Tumor Cells

**Figure 1:**
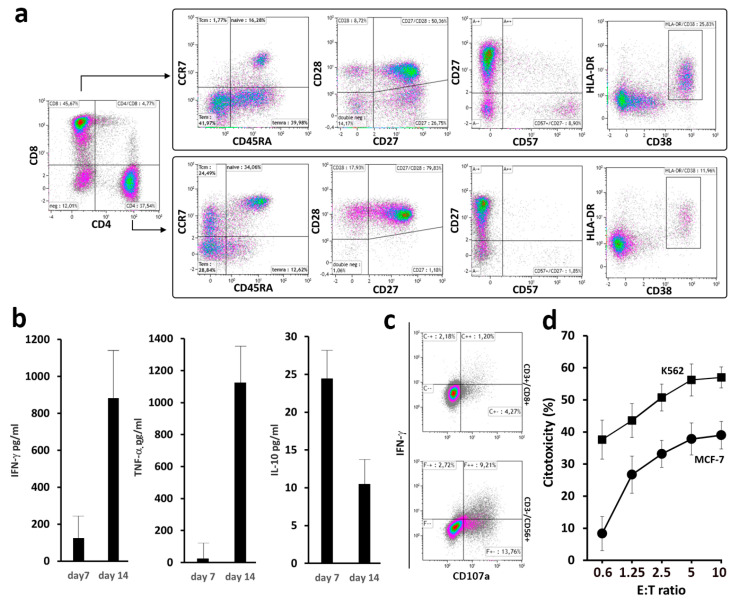
Phenotypic and functional analysis of peripheral blood lymphocytes (PBL) stimulated with HOCl-oxidized MCF-7 breast tumor cell lysate. (**a**) Dot-plot analysis of CD4+ and CD8+ cells as determined by flow cytometry in PBL stimulated with IFN-DC. PBL isolated from HLA-A2+ healthy blood donors were cultured with autologous IFN-DC (IFN-DC/PBL ratio of 1:4) pulsed with MCF-7 tumor cell lysate, as described in Section 2. Representative results of three independent experiments are shown. (**b**) Cytokine production (IFN-γ, TNF-α and IL-10) in culture supernatants as evaluated by ELISA on days 7 and 14 of co-culture. (**c**) Degranulation assay as a surrogate evaluation of cytotoxic activity by detection of CD107a membrane expression and intracellular IFN-γ production in CD8+ and natural killer (NK) cells. Representative dot-plot analysis in electronically gated CD8+CD3+ and CD56+CD3- cells in PBL co-cultured with tumor cell-loaded IFN-DC. On day 21 of culture, PBL were restimulated with MCF-7 or K562 target cell lines for 4 h at 37 °C (E:T ratio of 2:1) (see Section 2). Dot-plots show CD107a membrane exposure and IFN-γ expression in electronically gated CD8+CD3+ and CD56+CD3- lymphocytes in response to the indicated target cells. Results from one representative experiment out of four are shown. (**d**) Representative cytotoxicity assay against NK-sensitive K562 and MCF-7 target cell lines as evaluated by a Calcein-AM assay (see Section 2) at different E:T ratios. Data are mean ± SD of a triplicate assay of PBL derived from an HLA-A2+ donor.

A preliminary set of experiments was conducted to evaluate the overall technical feasibility and to ascertain the immune response elicited by our vaccine. This was achieved by loading IFN-DC from HLA-A2+ healthy donors with oxidized tumor cell lysate from HLA-matched MCF-7 breast tumor cells. The peripheral blood lymphocytes (PBL) cultured with tumor cell-loaded IFN-DC for 14 days exhibited activated and highly differentiated CD8+ cells, indicative of enhanced cytotoxic potential (Figure 1a). Significant levels of IFN-γ and TNF-α were detected in the culture supernatants by ELISA on days 7 and 14, indicative of a Th1-biased response (Figure 1b). Very low levels of IL-10, a Th2 and inhibitory cytokine, were detected on day 7 of culture, with a further decline observed in subsequent days of culture.

The antitumor activity was confirmed by a degranulation assay. As shown by specific CD107a membrane expression and IFN-γ production, MCF-7 breast tumor cells were effectively recognized by both CD8+ T lymphocytes and natural killer (NK) cells (Figure 1c). These data were consistent with the results of the cytotoxicity test (Figure 1d), demonstrating efficient MCF-7 cell killing. Improved K562 cell killing was also shown, indicating NK cell activation. This was not unexpected, as early IFN-γ production and NK cell activation induced by IFN-DC have been previously described in another tumor model [16].

### 3.2. In Vivo Efficacy of Therapeutic Vaccination with IFN-DC Loaded with HOCl-Oxidized Tumor Cell Lysate

**Figure 2:**
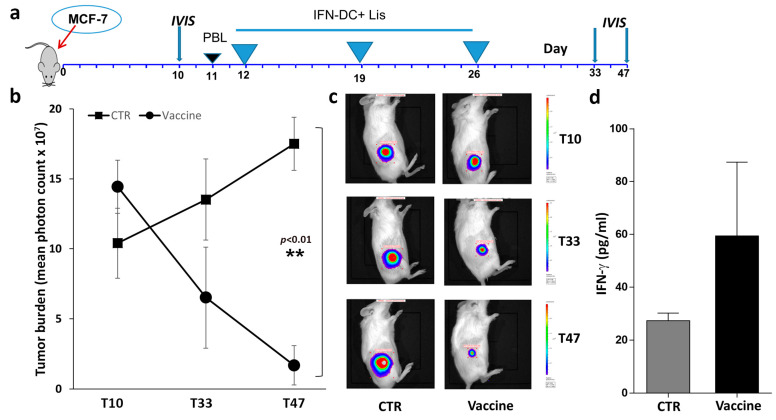
Evaluation of tumor-growth inhibition by in vivo immunization of hu-PBL-NSG mice with IFN-DC loaded with HOCl-oxidized tumor cell lysate. (**a**) Vaccination schedule. Mice were reconstituted with HLA-A2+ PBL as soon as the implanted tumors became detectable by in vivo bioluminescence imaging (10–11 days). Humanized mice were then randomized into treatment and control groups. (**b**) Quantitative evaluation of tumor cell growth by bioluminescence analysis. Tumor burden was detected by in vivo non-invasive imaging of the firefly luciferase expressing MCF-7 cells after intraperitoneal luciferin injection. Tumor bioluminescence intensity was plotted in pseudocolor over black/white photographs and quantified as total flux in photons/seconds. Graph represents mean total flux of MCF-7 cell growth rate in hu-PBL-NSG mice immunized as described. The data are presented as mean ± SEM. The difference in tumor growth was highly statistically significant only at the last time point, day 47 (** *p* < 0.01 by Mann–Whitney test). (**c**) Representative tumor burden images of the two groups (CTR vs. vaccine) at different time points by IVIS imaging system. (**d**) Evaluation of IFN-γ levels in mouse sera collected at the time of sacrifice.

We subsequently examined the potential of IFN-DC loaded with (HOCl)-oxidized MCF-7 cell lysate to impede tumor growth in vivo in a humanized NOD SCID gamma (NSG) mouse model. Mice were engrafted with MCF-7 tumor cells expressing the firefly luciferase reporter gene and reconstituted with human PBL from HLA-A2+ healthy donors. The humanized mice were randomly assigned to treatment and control groups (Figure 2a) and vaccinated three times at seven-day intervals via intraperitoneal administration of autologous IFN-DC loaded with HOCl-oxidized tumor cell lysate. Since the tumors frequently exhibited a relatively flat carpet-like growth pattern, the majority remained undetectable until they reached a considerable size. As demonstrated by bioluminescence imaging, the tumor cells exhibited rapid growth in the control mice, whereas vaccination resulted in tumor inhibition within three weeks of treatment, thus substantiating the efficacy of therapeutic vaccination (Figure 2b,c). At the time of sacrifice, vaccinated xenochimeric mice exhibited elevated levels of IFN-γ production in their serum (Figure 2d). The majority of control mice that received unloaded IFN-DC exhibited cachexia and required euthanasia, limiting the duration of the experiments to seven weeks.

### 3.3. Patient-Specific BC Organoids Allow an Efficient Expansion of BC Cells and a Faithful Reconstruction of Parental Tumors Maintaining Antigenic Profiles

**Figure 3:**
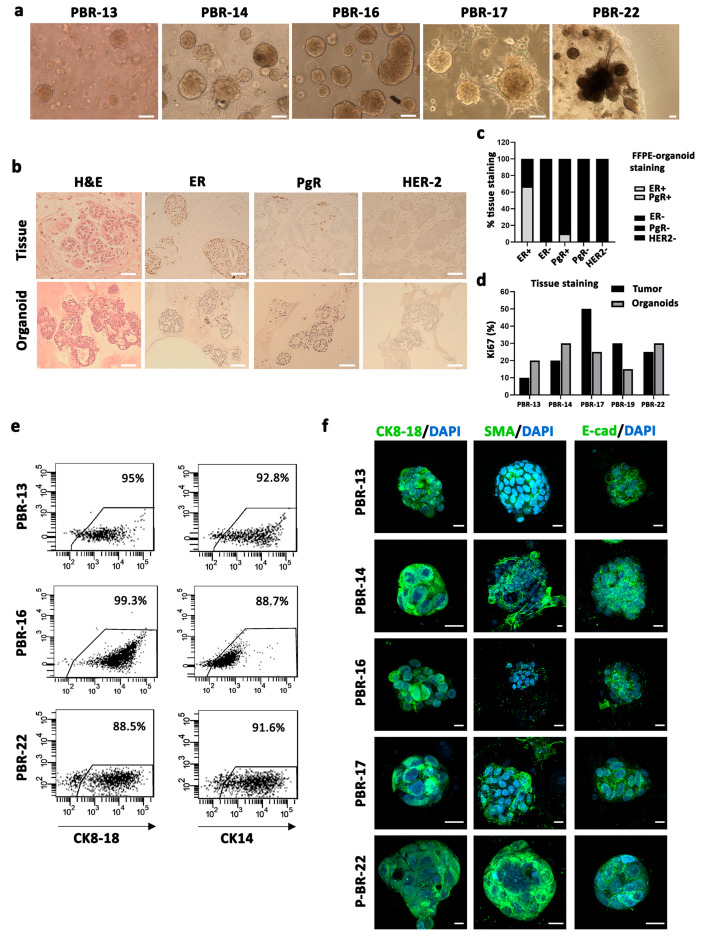
Isolation and characterization of patient-derived breast cancer organoids (PDBCOs). (**a**) Representative bright-field images of 5 PDBCOs used for the study, showing different structures: cohesive and discohesive organoids, dense and solid (PBR-13, PBR-14, PBR-16, PBR-17), cohesive organoids, cystic and grape-like (P-BR-22). Scale bar, 100 µm. (**b**) Comparative histological and immunohistochemical images of BC tissues and derived organoid lines. Shown are representative examples of H&E staining and IHC of either HR or HER-2 status for PBR-17. Scale bar, 200 µm. (**c**) Stacked bar chart indicating the percentage of PDBCO lines found positive (grey) and negative (black) by IHC for the receptor expression grouped per original tumor receptor status. (**d**) Bar graph displaying proliferation rate percentage of PDBCOs and corresponding parental tumors as quantified by Ki67 immunohistochemical staining. (**e**) Representative dot-plot graphs showing CK14 and CK8-18 expression in PBR-13, PBR-16 and PBR-22 (**f**) CLSM analyses of PFA-fixed PDBCOs stained for CK8-18, SMA and E-cadherin (green); 4′-6-Diamidino-2-phenylindole (DAPI) was used to counterstain nuclei (light blue). Several (>50 organoids) were observed for each condition and representative images are shown. Scale bars, 10 µm.

The primary challenge associated with cancer vaccination using autologous tumor cell lysate is the relatively low cell yield obtained from surgical samples, which is insufficient to produce the requisite amount of lysate for loading onto dendritic cells and conducting an adequate number of vaccination cycles. This limitation can be addressed through the expansion of cancer cells in vitro. However, the major drawback of this step is the possible alteration of the original tumor antigenic profile [18,19]. Three-dimensional (3D) tumor-derived organoids better mimic in vivo tissue and are regarded as a more realistic method for reproducing the antigenic profile of parental tumor cells. We obtained 19 tumor organoids from 22 BC specimens with different histological subtypes (invasive ductal carcinoma, invasive lobular carcinoma, neuroendocrine carcinoma) (Table 1), by seeding dissociated tumor-derived cells in matrigel drops covered with a BC organoid medium as described by Clevers et al. [15]. BC cells self-organized into multiple different structures generating both discohesive and cohesive solid, cystic or grape-like organoids (Figure 3a). We selected five patient-derived BC organoids (PDBCOs), which could be long-term expanded (>15 passages). Histological analysis confirmed that PDBCOs maintained the morphological and structural features of primary tumors, showing ductal-like patterns and cribriform architecture (Figure 3b). In the majority of PDBCO cultures, the expression of estrogen receptor (ER) and HER-2 correlated with parental tumors (Figure 3c), although there was a reduction in the expression levels, probably due to an insufficient ERα signaling stimulation [20,21,22]. PDBCOs largely maintained a sustained proliferation rate (>15%) in culture, as quantified by Ki67 immunostaining (Figure 3d) and preserved a mammary epithelial phenotype, confirmed by lineage-specific marker expression such as the luminal cytokeratins (CK) 8, 18 or 19 or E-cadherin and the basal–myoepithelial cytokeratins 5 and 14, smooth muscle actin (SMA) or the P63 protein [23] (Figure 3e,f, Figures S1b and S2a,b). In particular, confocal laser scanning microscope (CLSM) analyses revealed that CK8/18 is highly expressed throughout the cytoplasm, as is the SMA marker; whereas the expression of E-cadherin was almost exclusively confined to the plasma membrane (Figure 3f), as indicated by the typical punctuate pattern. Moreover, CLSM analysis on PDBCOs confirmed their pathologic origin showing high expression levels of epithelial–mesenchymal transition (EMT) markers, such as vimentin, and proteins associated with tumor aggressiveness and invasiveness, such as TAZ and β-catenin (Supplementary Figure S1a,b) [24,25,26]. Furthermore, as highlighted by the double staining with CK8/18 and SMA (3D reconstruction in Supplementary Figure S1b), these PDBCOs contained progenitor cells, in a transitional state, where they simultaneously expressed CK8/18 and SMA. These cells fully differentiate into secretary luminal cells (CK18/8+) and myoepithelial cells (SMA+), indicating that the putative stem cells are in the luminal/suprabasal compartment [27,28]. Both cytofluorimetric and immunohistochemistry (IHC) analysis highlighted the presence of a subpopulation expressing the putative BC stem cell immunophenotype (CD44+/high/CD24−/low), responsible for tumor maintenance and relapse [29] (Supplementary Figure S2a–c), suggesting that primary tumor-cell heterogeneity is preserved in organoids.

**Figure 4:**
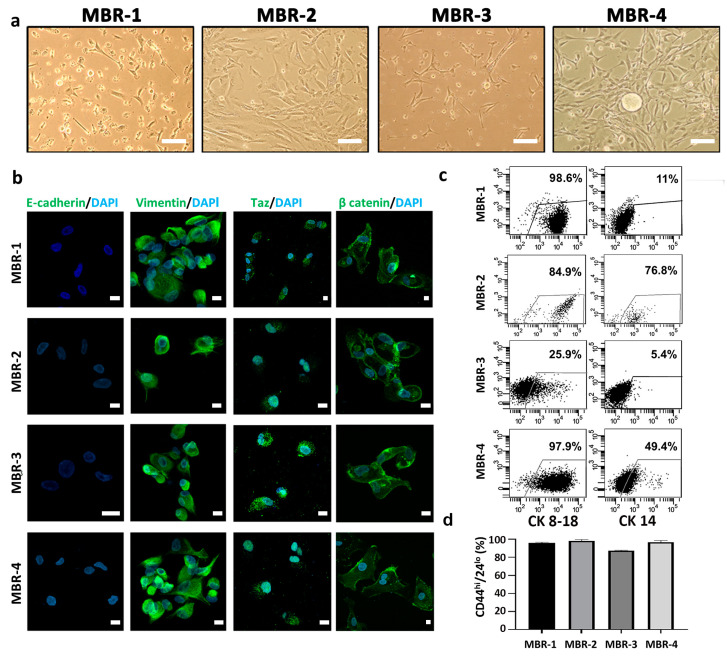
Isolation and characterization of breast cancer patient-derived metastatic cells (PDMCs). (**a**) Representative phase-contrast images of PDMCs from ascitic fluid (MBR-1, MBR-2) or pleural effusion (MBR-3, MBR-4) cultured in serum-free conditions. Scale bar, 100 µm. (**b**) CLSM analyses of PFA-fixed PDMCs stained for E-cadherin, vimentin Taz and beta-catenin (green); DAPI was used to counterstain nuclei (light blue). Several fields were observed for each condition and representative images are shown. Scale bars, 10 µm. (**c**) Dot-plots showing luminal CK8-18 and myoepithelial CK14 expression in PDMC lines. (**d**) Bar chart reporting percentages of CD44^high^CD24^−/low^ phenotype in MBR-1, MBR-2, MBR-3 and MBR-4 lines obtained from ascitic fluid or pleural effusion of breast cancer metastatic patients. Percentages, referring to CD44^high^CD24^−/low^ positive cells, were determined by setting the gate on the isotype control from at least two independent FACS stainings.

We also isolated patient-derived metastatic BC (MBR) cells from ascitic fluid (n = 2, called MBR-1, MBR-2) or pleural effusion (n = 2, called MBR-3, MBR-4) of patients undergoing paracentesis or thoracentesis, respectively (Figure 4a). ER, PgR, and HER2 analysis confirmed that MBRs were triple-negative, as were the parental BC metastatic lesions. CLSM analyses revealed that all MBR cells underwent EMT. Indeed, all samples lost the E-cadherin expression, while becoming positive for Vimentin (Figure 4b). In addition, they displayed nuclear Taz localization and cytosolic accumulation of β-catenin usually related to the high aggressiveness in BC [14,24,30] (Figure 4b). Furthermore, Wilms Tumor 1 (WT1) IHC staining confirmed the association of MBR cells with a mesenchymal phenotype [24,30,31] (Supplementary Figure S3b). Cytofluorimetric analysis for CK8-18 and CK14 validated the mammary origin of MBR cells (Figure 4c). Due to the important role of CSCs in promoting metastatic dissemination in solid tumors [14], we investigated the presence of CD44high/CD24low populations in our MBR cells. FACS and IHC staining revealed an enrichment of CSCs (Figure 4d, Supplementary Figure S3b) and high PD-L1 expression in all MBRs (Supplementary Figure S3a).

### 3.4. In Vitro Evaluation of the IFN-DC-Based Vaccine in a Selected Group of Breast Cancer Patients

The peripheral blood of BC patients was analyzed by flow cytometry, confirming that that metastatic BC patients exhibit an altered immune phenotype, compared to the normal range of the standard peripheral blood parameters, involving both myeloid and lymphoid compartments, while primary tumor patients generally maintain physiological levels of the main circulating immune cells (Supplementary Figure S4).

**Figure 5:**
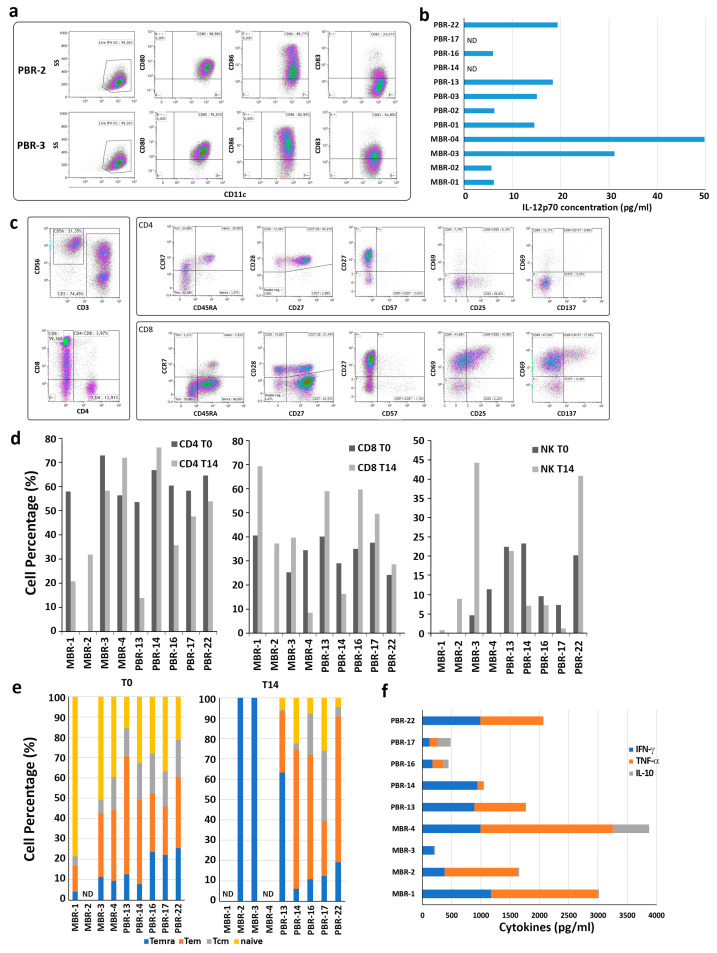
Characterization of PBL cultures from breast tumor patients stimulated with IFN-DC loaded with HOCl-oxidized autologous tumor cell lysate. (**a**). Representative phenotypical analysis of IFN-DC obtained from breast tumor patients. IFN-DC were differentiated from peripheral blood monocytes as described in Section 2. Partially mature CD11c+ IFN-DC ex-pressed high levels of the costimulatory molecules CD80 and CD86, as well as variable levels of the maturation marker CD83. (**b**) IL-12 release in supernatants collected from IFN-DC cultures on day 3 of differentiation. (**c**) Representative phenotypic analysis of PBL isolated from breast tumor patients and cultured with autologous IFN-DC loaded with HOCl-oxidized tumor cell lysate for 14 days. (**d**) Evaluation of CD4, CD8 and NK cell percentages in PBL from breast cancer patients before (T0) and after 14 days of culture (T14) with autologous IFN-DC. (**e**) Flow cytometric analysis of CD4 and CD8 cell memory subsets of freshly purified PBL from breast cancer patients and after 14 days of culture with autologous IFN-DC loaded with HOCl-oxidized tumor cell lysate. (**f**) Cytokine release (IFN-γ, TNF-α and IL-10) in culture supernatants as evaluated by ELISA on day 14 of co-cultures.

A standard monocyte culture with GM-CSF and IFN-α was used to differentiate IFN-DC [11], with the final cellular yield representing approximately 50% of the initial cell number. This outcome was fully comparable to that routinely obtained from healthy donors in terms of morphology and viability. Due to the limited number of monocytes that could be obtained from the patients’ blood samples, it was only possible to carry out phenotypic characterization of IFN-DC when the cellular yield was sufficiently high. The expression of the costimulatory markers CD80 and CD86, as well as the maturation marker CD83, was found to fall within the normal range (Figure 5a), indicating that differentiation was not altered. The culture supernatants of IFN-DC exhibited the presence of IL-12p70 (Figure 5b). In general, IFN-DC could be generated from all patients tested, loaded with HOCl-oxidized tumor cell lysates, and cryopreserved in ready-to-use aliquots. However, not all tumor samples obtained from BC patients could be expanded to an adequate number for IFN-DC loading in two cycles of in vitro stimulation.

Phenotypic analysis of the cultures demonstrated that CD8 T cells were preferentially expanded by tumor cell-loaded autologous IFN-DC (Figure 5c,d), with the exception of MBR-4 and PBR-14, which exhibited an outgrowth of CD4 T cells. The percentage of NK cells present in the cultures exhibited considerable variation. CD8 cells exhibited the hallmarks of highly differentiated subpopulations, predominantly Tcm and Temra (Figure 5e). The analysis of culture supernatants revealed a considerable degree of variability in IFN-γ and TNF-α production among the different BC patient donors. Of note, negligible amounts of the cytokine IL-10, which is known to hinder anti-tumor response, were detected in all cultures that had been stimulated with IFN-DC (Figure 5f).

**Figure 6:**
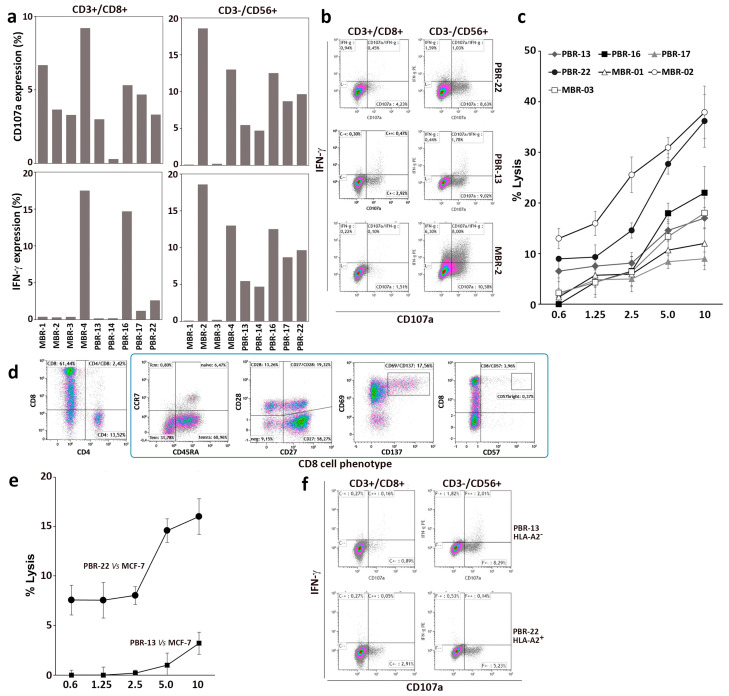
Antitumor activity of PBL from breast cancer patients co-cultured with IFN-DC loaded with HOCl-oxidized autologous tumor cell lysate. PBL isolated from nine patient blood donors were cultured with IFN-DC pulsed with HOCl-oxidized tumor cell lysate for 14 days. (**a**) Degranulation activity of expanded effectors cells tested against autologous breast cancer target cells as detected by flow cytometry; analysis of CD107a membrane expression and intracellular IFN-γ. (**b**) Representative degranulation assay as assessed by dot-plot analysis of ectopic CD107a and IFN-γ expression in CD8+ and CD3-CD56+ NK cells derived from three representative breast cancer patients in response to autologous tumor cells. (**c**) Cytotoxic assay of PBL culture from breast cancer patients after in vitro culture for 14 days, tested against autologous breast cancer cells. (**d**) Phenotypic analysis of CD8 cells from patient PBR-22 (HLA-A2+) stimulated with IFN-DC loaded with HOCl-oxidized autologous tumor cell lysate for 14 days. (**e**) Cytotoxic activity of PBL from PBR-22 (HLA-A2+) as compared to PBR-13 (HLA-A2–) as determined by cytotoxic assay towards HLA-A2+ MCF-7 target cells. (**f**) Degranulation activity as determined by dot plot analysis of CD107a and IFN-γ expression in CD8+ and CD3-CD56+ NK cells from patients PBR-22 and P-BR13 toward MCF-7 target cells.

The immune effector response to autologous BC cells was determined. The degranulating activity of CD8 and NK cells in response to BC target cells was evaluated by flow cytometry, together with the expression of IFN-γ (Figure 6a,b). All PBL cultures from individual patients stimulated with oxidized lysate-loaded IFN-DC demonstrated an antitumor response, as evidenced by degranulation and IFN-γ production against autologous tumor cells. In accordance with the experiments performed with healthy donor PBL and MCF-7 cells, a contribution to the anti-tumor response was provided by CD3-/CD56+ NK cells, which exhibited vigorous degranulation activity and IFN-γ production in response to primary breast tumor cells. The specific tumoricidal activity was evaluated by a Calcein-AM cytotoxic assay, which demonstrated the efficient killing of autologous tumor targets at each effector-to-target (E:T) cell ratio (Figure 6c). This suggests that oxidized breast tumor cell lysate loaded onto IFN-DC can effectively elicit anti-tumor effector cells to kill breast tumor cells in vitro.

Noteworthy, the stimulated lymphocytes from patient PBR-22, who exhibited HLA-A2 positivity, demonstrated the capacity to recognize and kill HLA-matched MCF-7 BC cells. However, the efficacy was comparatively lower than that observed with the autologous tumor (Figure 6b,c). In contrast, control HLA-A2 mismatched lymphocytes from patient PBR-13 exhibited minimal lytic activity (Figure 6e) but demonstrated nonspecific NK activity against MCF-7 cells (Figure 6f).

## 4. Discussion

Breast cancer (BC) has historically been considered a challenging malignancy to treat with immune checkpoint inhibitors, largely due to its intrinsic low immunogenicity. Nevertheless, the potential therapeutic applications of immunotherapy in patients with breast tumors have recently been the subject of renewed interest [32,33]. Furthermore, recent clinical phase I and II trials have demonstrated the efficacy and safety of DC vaccines, particularly in combination regimens, as a promising approach to BC treatment. These studies indicate that dendritic cell (DC)-based vaccines have the potential to restore and enhance immune responses, as well as prolong patient survival [34,35,36,37]. It is noteworthy that there is currently no general consensus regarding the optimal type of DC to be used, and the most effective antigen formulation remains an unresolved issue. However, it appears that the most effective personalized breast cancer vaccines should be tailored to individual tumors [38,39], given the significant challenges posed by tumor heterogeneity, which represents a major barrier to treating this cancer [38,40]. A recent advance to address the aforementioned issue in personalized cancer vaccines has been the use of neoantigen-containing peptides, predicted through a bioinformatics platform following tumor genome sequencing [41]. An alternative strategy has been anticipated, whereby overexpressed survivin is proposed as a universal vaccine target in malignancies such as breast cancer [42]. Nevertheless, the in vitro efficacy of an autologous DC vaccine has been demonstrated in a small cohort of BC patients. In this approach, primary tumor cells were expanded in vitro for several weeks and used as a cell lysate to load DC [43].

Our recent findings indicate that IFN-DC loaded ex vivo or in vivo with whole-tumor cell lysates are promising candidates for cancer vaccination, particularly effective in cross-presentation of tumor-associated antigens to CD8 T-cells and in their expansion [17,44,45]. In this study, we introduce a novel cancer vaccine based on a lysate derived from cultured tumor organoids loaded onto autologous IFN-DC. This vaccine is capable of inducing robust immune responses against patient-derived BC cells. It is important to note that the primary cancer cells utilized in this study were obtained from individual breast tumors with distinct histological features, making the vaccine highly targeted to the specific type of cancer present in each patient. Indeed, patient-derived BC organoid cultures faithfully replicate the biological and antigenic characteristics of primary breast tumors in vitro, making them an appropriate antigenic source.

We first assessed the efficacy of an IFN-DC-based vaccination with oxidized tumor cell lysate in eliciting a strong antitumor response against the MCF-7 BC cell line. The approach was evaluated in two distinct settings: in vitro and in a xenochimeric hu-PBL-NSG mouse model. In the in vitro setting, the vaccination regimen was found to induce regression of pre-established MCF-7 breast cell tumors when administered 10–11 days after tumor implantation. Furthermore, we demonstrated that monocyte-derived IFN-DC loaded with oxidized tumor cell lysate derived from both patient-derived metastatic cells and BC organoids can effectively elicit an anti-tumor Th1-skewed response and CD8⁺ cytotoxic activity. This is capable of effectively killing autologous cancer cells in vitro, as determined by degranulation tests and cytotoxicity assays. Notwithstanding the altered immune profile observed in metastatic BC patients involving both myeloid and lymphoid compartments, we could also demonstrate the regeneration of an anti-tumor response in vitro.

Given the intrinsic heterogeneity of BC, the development of patient-specific autologous vaccines may be of critical importance. In this regard, the IFN-DC loaded with antigens from BC patient-derived organoids treated with hypochlorous acid proved to be an optimal antigenic formulation for the development of a personalized breast cancer vaccine, effectively addressing the issue of tumor heterogeneity. Moreover, while a defective differentiation and functional alteration of the endogenous DC has been observed in cancer patients [46], our results indicate that the generation of autologous IFN-DC ex vivo may potentially circumvent tumor-induced dysfunction in antigen-presenting cells. PDBCOs carry a broad range of tumor-associated antigens, thereby increasing the likelihood of eliciting a robust immune response against cancer cells. Moreover, in contrast to conventional treatments such as chemotherapy, DC vaccines are typically associated with a reduced incidence of adverse effects and lower toxicity levels [47].

It is important to note that while the use of DC vaccines derived from organoids holds promise, further research and clinical trials are necessary to validate their efficacy and safety. In this regard, further studies will be required to improve cell yield from organoid culture and to determine optimized vaccine administration methods, immunization schedules, and timing of vaccination boosts necessary to maintain a durable immune response critical to prevent tumor relapse.

## 5. Conclusions

The present study demonstrates the efficacy of a candidate cancer vaccine based on IFN-DC loaded with breast tumor organoids and points toward the potential of oxidized cell lysate to enhance tumor immunogenicity. This work is important, as this approach is promising for personalized immunotherapy of breast cancer, particularly in the neo-adjuvant setting, after surgical resection, to prevent tumor relapse. However, to achieve a synergistic effect for cancer eradication, we envisage that effective future clinical application of breast cancer vaccines will also rely on appropriate combinations with tumor-targeting monoclonal antibodies, tyrosine kinase inhibitors, chemotherapeutics and immune checkpoint inhibitors. In particular, since the clinical efficacy of immune checkpoint inhibition depends on the extent of specific T-cell infiltration in tumor tissues, our vaccine candidate may prove an effective adjunct to immune checkpoint blocking therapy [48]. Conversely, IFN-γ production by infiltrating CD8+ T cells has been shown to upregulate PD-L1, indoleamine 2,3-dioxygenase (IDO) and Tregs in the tumor microenvironment [49,50], contributing to impair T cell responses and dampening vaccine efficacy [51]. Thus, as a logical extension of our research, we propose evaluating the potential benefits of combining the vaccine with selected immune checkpoint inhibitors that interfere with the PD-1/PD-L1 axes, such as pembrolizumab and atezolizumab [32,33,34], as a strategy to reverse immunosuppressive signals mediating resistance to antitumor vaccination.

## Figures and Tables

**Table 1:** Clinicopathological characteristics of (**a**) primary breast cancer patients and (**b**) metastatic breast cancer patients.

**(a)**									
**Patient** **No.**	**Age**	**Menopausal** **Status**	**Histology**	**Pathology,** **Tumor Size** **(pT)**	**Lymph Node** **Status (pN)**	**Grade**	**ER/PgR/HER2 ^2^** **Status (%)**	**Ki67 (%)**	**Neo-Adjuvant** **Treatment and Response**
1	37	Pre	IDC nos ^1^	T2	SN neg	3	-/-/-	70	NA ^3^
2	69	Post	ILC	T1b(m)	N2a	X ^4^	95/1/-	5	Ctx-Epi + Txl(RP)
3	46	Pre	NET	T2	N1(sn)	3	80/80/-	70	NA
4	34	Pre	IDC	T2ypM1(L)	N1a	3	-/-/-	60	Beva + Txl (RP-Li; PD-T)
5	52	Post	IDC	T2	N1(mi)	2	90/2/-	30	NA
6	73	Post	IDC	T2	N0(sn)	2	90/3/-	25	NA
7	75	post	IDC nos	T1c	N1mi(sn)	2	95/95/-	20	NA
8	82	Post	IDC	T3	N0(sn)	2	95/95/-	20	NA
9	49	Pre	R ^5^:IDC	T2	N0(sn)	2	95/95/-	30	NA
			L: ILC	T2m	N1(sn)	2	95/95/-	15	
10	62	Post	IDC	T1c	N1mi(sn)	2	95/95/-	20	NA
11	43	Pre	IDC	T1c	N1a	2	95/95/-	20	NA
12	46	Pre	IDC nos	T2	N0(sn)	2	95/95/-	20	NA
13	49	Pre	IDC nos	T1c	N1a	2	90/90/-	10	NA
14	51	Pre	Ca papillary + NET	T2	N1(sn)	2	95/85/-	20	NA
15	66	Post	IDC	T2	N0(sn)	3	10/-/-	50	NA
16	50	Pre	ILC + NET	T3	N1a	3	90/90/-	50	NA
17	32	Pre	IDC apocrine	T2(m)	N1a	3	80/-/-	30	NA
18	76	Post	IDC	T1c(m)	N3a	3	-/-/-	25	NA
19	41	Pre	IDC	T2	N0	3	-/-/-	80	Epi + Taxol(PD)
20	78	Post	IDC nos	T2	N1mi(sn)	2	95/70/-	15	NA
21	79	Post	IDC nos	T2(m)	N1a	2	95/-/-	22	NA
22	77	Post	ILC	T2	N1a	2	90/40/-	25	NA
^1^ IDC, infiltrating ductal carcinoma; DCIS, ductal carcinoma in situ; ILC, invasive lobular carcinoma; ILCI, lobular carcinoma in situ, Neuroendocrine tumor (NET); sn (sentinel lymph node); mi (micrometastasis); ^2^ ER, estrogen receptor; PgR, progesterone receptor, expressed in percentage; HER2status + positive or—negative; ^3^ NA, not applicable; Ctx: cyclophosphamide; Epi, epirubicin; Beva, bevacizumab; Txl: taxol; FEC, 5-fluorouracil, Epi, epirubicin, Ctx, cyclophoasphamide; ^4^ X, undetermined; ^5^ R: right side; L: left side.									
**(b)**									
**Patient** **No.**	**Age**	**Source of Cells**	**Histology** **on Original T**	**Pathology,** **pTNM Original T**	**Grade**	**ER/PgR/HER2 ^2^** **Status of the Original T (%)**	**ER/PgR/HER2** **Status of M Disease**	**Previous** **Treatments**	
1	50	Ascites	ILC ^1^	NA ^3^	NA	95/95/+	-/-/-	^5^ CMF, Herc, Tam, AI, Pertuzumab, Dxt, CDDP, Gem	
2	59	Ascites	DCIS+ comedoCa	pT1mN0 (m)	3	70/60/-	-/-/-	Fulvestrant, Everolimus, Exem; Txl	
3	67	PE ^6^	IDC	T3N+	3	95/30/-	-/-/-	NA (met at the diagnosis)	
4	43	PE	IDC	pT1cN1a	2	80/50/+	-/-/-	FEC, Herc, Cape, Lapatinb, Txl, CBDCA, CMF	
^1^ IDC, infiltrating ductal carcinoma; DCIS, ductal carcinoma in situ; ILC, invasive lobular carcinoma; ILCI, lobular carcinoma in situ comedoca, comedocarcinoma; Neuroendocrine tumor (NET); sn (sentinel lymph node); mi (micrometastasis); ^2^ ER, estrogen receptor; PgR, progesterone receptor, expressed in percentage; HER2status + positive or—negative; ^3^ NA, not applicable; Ctx: cyclophosphamide; Epi, epirubicin; Beva, bevacizumab; Txl: taxol; FEC, 5-fluorouracil, Epi, epirubicin, Ctx, cyclophoasphamide; ^4^ X, undetermined; ^5^ R: right side; L: left side1 ^5^ CMF, cyclophosphamide, methotrexate, 5-fluorouracil; Herc, Herceptin; Tam, Tamoxifen; A.I.: aromatase inhibitors; Exeme, exemestane; Dxt, Docetaxel; CDDP, Cisplatino; GEM, gemcitabine; Txl, Taxol; Cape: capecitabina; CBDCA: carboplatino; ^6^ PE: pleural effusion.									

## Data Availability

Data are available on reasonable request.

## Supplementary Materials

The following supporting information can be downloaded at: https://www.mdpi.com/article/10.3390/vaccines12091058/s1, Figure S1: CLSM analyses of PFA-fixed PDBCOs; Figure S2: Breast tumor marker expression; Figure S3: PD-L1 expression in metastatic cells derived from ascitic fluid or pleural effusion of metastatic breast cancer patients, Figure S4: Frequency of immune cell subsets in primary, metastatic patients and healthy female volunteers.
