# Supplementary material for: Anti-Tumor Immunity to Patient-Derived Breast Cancer Cells by Vaccination with Interferon-Alpha-Conditioned Dendritic Cells (IFN-DC)
Source: Vaccines (Basel). 2024 Sep 17;12(9):1058. doi: 10.3390/vaccines12091058 (PMC11435915; doi:10.3390/vaccines12091058)
Supplement: Supplementary file 1 [file vaccines-12-01058-s001.zip › vaccines-3168394-supplementary.pdf]

Supplementary figures

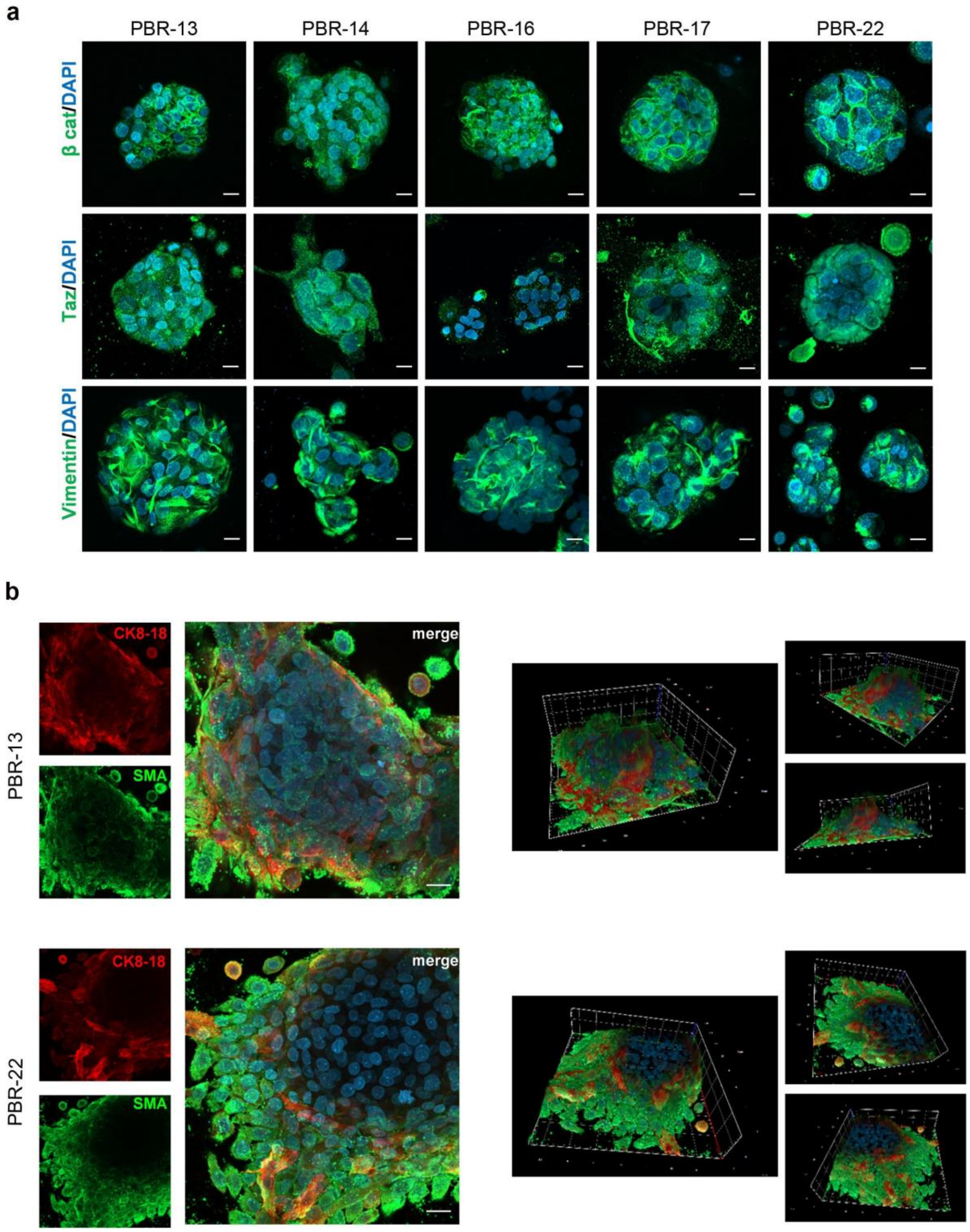

Figure S1. CLSM analyses of PFA-fixed PDBCOs stained for b-catenin, TAZ, vimentin (green). DAPI was used to counterstain nuclei (light blue). Several (>50) organoids were observed for each condition and representative images are shown. Scale bars, 10 µm. (B) CLSM analyses of PFA-fixed PDBCOs (PBR-13 and PBR-22) double stained for CK8-18 (red) and SMA (green). DAPI was used to counterstain nuclei (light blue). Several (>50) organoids were observed for each condition and representative images are shown. Scale bars, 20 µm.

a

FFPE-ORGANOIDS	CD44 (%)	CD24 (%)	CK19 (%)	CK5 (%)	P63 (%)	VIM (%)
**P-BR-13**	95	20	95	95	5	40
**P-BR-14**	95	10	40	90	40	70
**P-BR-16**	100	0	35	90	2	70
**P-BR-17**	95	0	90	100	0	0
**P-BR-18**	80	0	20	90	0	70
**P-BR-19**	95	0	30	95	30	80
**P-BR-22**	90	0	60	95	0	70

b

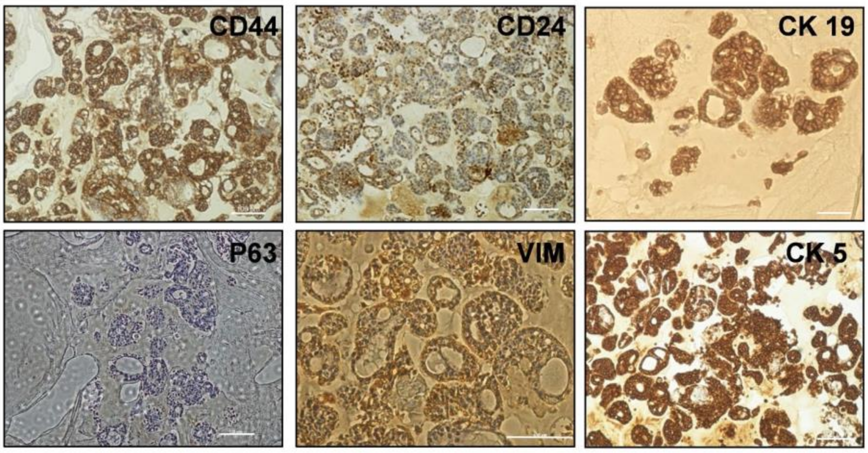

c

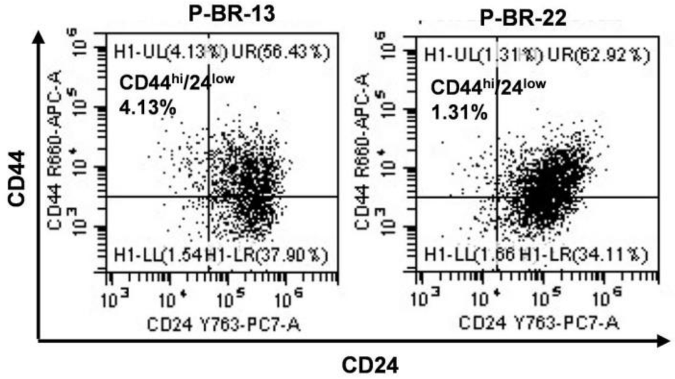

d

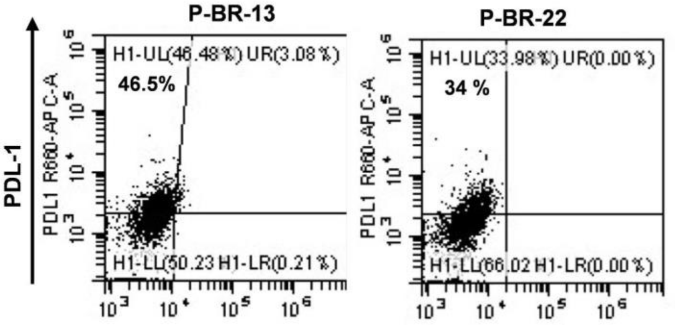

Figure S2. Breast tumor marker expression. (a) Table listing breast tumor marker expression evaluated by immunohistochemical staining on FFPE-PDBCOs. (b) Representative IHC staining for the tumoral markers listed on table in Supplementary Figure 2a. (c) Cytofluorimetric analysis of CD44/CD24 double staining in PBR-13 and PBR-22 obtained from human breast primary tumors. Percentages, referring to CD44highCD24-/low immunophenotype, were determined by setting the gate on the isotype control. (d) Dot-plot graphs showing PD-L1 expression in PBR-13 and PBR- 22 organoids. Percentages were determined by setting the gate on the isotype control.

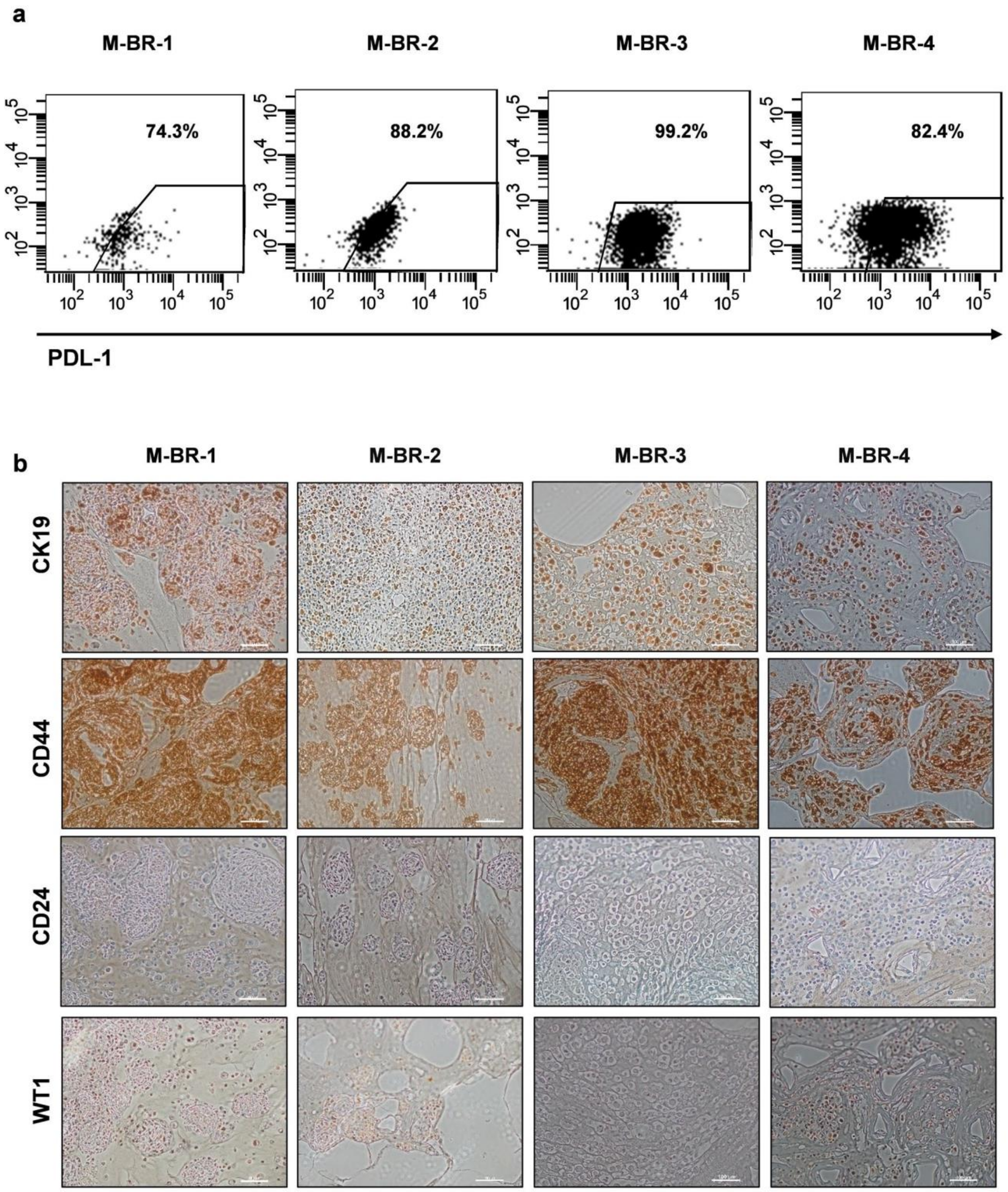

Figure S3. PD-L1 expression in metastatic cells derived from ascitic fluid or pleural effusion of metastatic breast cancer patients. (a) Dot-plot graphs showing PD-L1 expression in metastatic cells derived from ascitic fluid or pleural effusion of metastatic breast cancer patients. Percentages were determined by setting the gate on the isotype control. (b) Representative IHC staining on FFPE-MBRs for CK 19, CD44, CD24 and WT1.

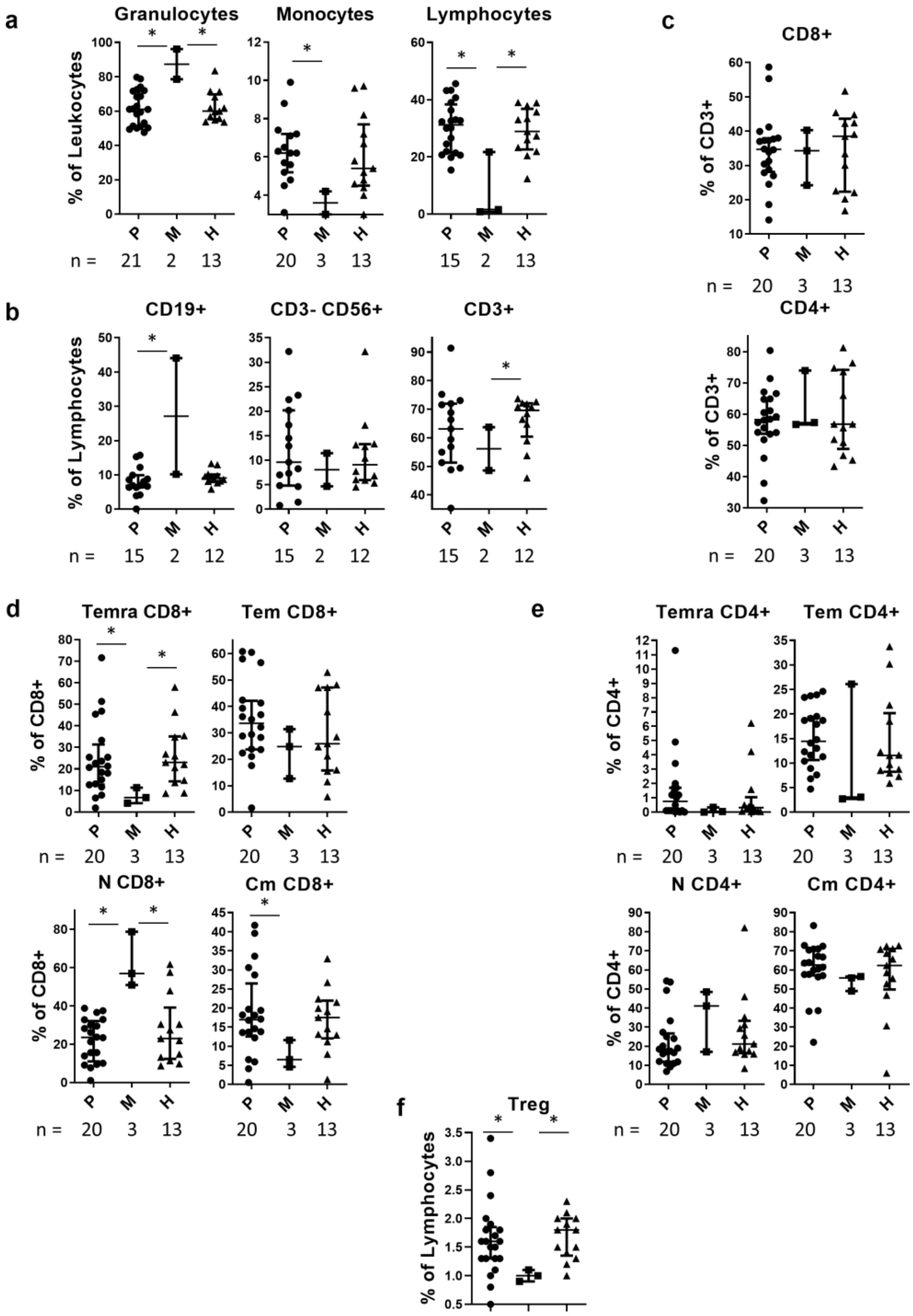

Figure S4. Frequency of immune cell subsets in primary, metastatic patients and healthy female volunteers. Scatter plots (showing single values, median and interquartile range) represent *ex-vivo* frequencies of different circulating immune cell subsets. (a) Major leukocyte subpopulations (granulocytes, lymphocytes and monocytes) expressed as a percentage of leukocyte gated cells; (b) Percentages of main cellular subsets within lymphocyte gate including CD19+ B cells, CD3-CD56+ NK cells and CD3+ T cells; (c) CD3+CD4+ and CD3+CD8+ T cell percentages (expressed as % within CD3+ T cell gate). Naïve/memory T cell subsets based on CCR7 and CD45RA expression within (d) CD8+ and (e) CD4+ T cell gate. f) Regulatory T cell (Tregs, CD3^{+}CD4^{+}CD25^{hi}CD127^{-}Foxp3^{+}) percentage within lymphocyte gate. * = p values < 0.050, obtained by non-parametric unpaired Mann-Whitney U-test.
